# Mast cells as important regulators in the development of psoriasis

**DOI:** 10.3389/fimmu.2022.1022986

**Published:** 2022-11-03

**Authors:** Xu-Yue Zhou, Kun Chen, Jia-An Zhang

**Affiliations:** Institute of Dermatology, Jiangsu Key Laboratory of Molecular Biology for Skin Diseases and STIs, Chinese Academy of Medical Science and Peking Union Medical College, Nanjing, China

**Keywords:** mast cell, psoriasis, T cell, cell network, inflammation

## Abstract

Psoriasis is a chronic inflammatory immune skin disease mediated by genetic and environmental factors. As a bridge between innate and adaptive immunity, mast cells are involved in the initiation, development, and maintenance of psoriasis by interactions and communication with a variety of cells. The current review describes interactions of mast cells with T cells, Tregs, keratinocytes, adipocytes, and sensory neurons in psoriasis to emphasize the important role of mast cell-centered cell networks in psoriasis.

## Introduction

Psoriasis is a chronic-relapsing inflammatory autoimmune skin disorder mediated by complex interactions between genetic and environmental factors. Psoriasis can be triggered by injury or infection, and is characterized by the abnormal proliferation and differentiation of keratinocytes and infiltration of inflammatory cells, including T cells, mast cells (MCs), neutrophils, and macrophages. Although the pathogenesis of psoriasis is not fully understood, the dysfunction of innate and adaptive immune, especially the IL-23/IL-17 pathogenic axis, is now considered to play key roles in the development of psoriasis (1).

MCs are tissue-resident immune cells. Upon maturation, they are primarily located in barrier tissues, such as the skin, respiratory tract, and intestinal mucosa. The skin is highly enriched in MCs, which account for 8% of the total number of cells in the dermis, located near the epidermis and the subcutaneous vascular system and nerves (2). As local tissue sentinels, MCs are often initially activated by external environmental stimuli or pathogen invasion and subsequently release a variety of mediators, including *de novo* synthesized cytokines and chemokines and pre-stored histamine, proteases, leukotrienes, and prostaglandins in cytoplasmic granules, thus exerting immunomodulatory functions or driving allergic responses (3). MCs are key initiators and regulators of innate and adaptive immunity, and their complex communication with other cells is involved in the maintenance of barrier function and immune homeostasis (4). Several studies have confirmed the important role of MCs in regulating epidermal barrier function and maintaining immune homeostasis in the skin, including roles in wound healing and processes related to skin aging (5, 6). Moreover, MCs are required in allergen-induced skin inflammation. DC immunoreceptor (DCIR) is upregulated in skin MCs of patients with atopic dermatitis and mediates allergen binding and uptake (7). Thus, MCs have a strategic position in host defense, the response to allergens, and immune homeostasis.

## Mast cells in psoriasis

Toruniowa et al. found that the number and degranulation of MCs were higher in the early stages of psoriasis lesions, especially in newly formed psoriatic lesions, than in mature psoriatic lesions. The Koebner phenomenon refers to the occurrence of new psoriatic lesions in the area of healthy skin following traumatic injury to patients with psoriasis. There is a consistent trend towards an increase in MCs in Koebner’s isomorphic response (8). The number of MCs in psoriatic lesions is reduced by successful treatment with anthralin, psoralen plus ultraviolet A irradiation (PUVA), or cyclosporine (9–11).

FcϵRI is a high-affinity IgE receptor that is constitutively expressed on MCs. IgE can recognize exogenous antigens (Ags), facilitate antigen presentation *via* FcϵRI, trigger the degranulation of MCs and the release of multiple mediators, and regulate innate and acquired immunity. Several reports have shown that total serum IgE levels are significantly elevated in patients with psoriasis and that IgE^+^ FcϵRI^+^ cells are significantly increased in psoriatic lesions and decrease with the improvement of the disease (12, 13). A non-classical IgE response may occur in psoriasis. MCs can directly bind to immature dendritic cells (DCs), induce increased expression levels of the costimulators CD86, CD80, and CD40 on the DC surface, indicating DC maturation, and promote the release of T cell-modulating cytokines, such as IFN-γ, IL-12, IL-6, and TGF-β, enhancing the ability of DCs to induce the proliferation of CD4+ T cells and Th1 and Th17 polarization (14). Increased IL-17A induces IgG memory B cells to produce IgE by class-switch recombination, forming immune complexes containing soluble IgE with psoriasis-associated autoantigens; these are presented to plasmacytoid (p) DCs *via* FcϵRI, activating pDCs to trigger inflammatory cytokine responses, including IFN-α, IL-6, IL-8, and TNF, involved in the development and maintenance of inflammatory processes in psoriatic lesions (15–17). Future studies are needed to determine how IgE-FcϵRI interactions precisely affect the activation of multiple immune cells in psoriatic skin lesions. Tryptases and chymases are classical mediators secreted by activated MCs and contribute to the persistence of inflammation by activating many innate and adaptive immune responses *via* protease-activated receptor 2 (PAR-2) (18). Serum tryptase levels are elevated in psoriasis (19). Tryptases can induce the production and release of multiple pro-inflammatory cytokines that directly cause and amplify psoriasis-associated inflammatory processes, such as IL-1β, IL-8, TNFα, and IL-6 production (20), and activate matrix metalloproteinases (MMP) to destroy various matrix components associated with the development of psoriatic arthritis (21). Pso p27 is an autoantigen detectable in MCs of psoriatic lesions and is thought to be produced by the non-canonical cleavage of MC chymases (22).

CIBERSORT is an analytical tool that uses a deconvolution algorithm to estimate the composition and abundance of various types of immune cells in samples based on gene-expression data (23). Several recent studies have used the CIBERSORT algorithm to assess immune cell infiltration in psoriasis. CIBERSORT uses a leukocyte gene signature matrix termed LM22 as the input matrix of reference gene expression signatures, with 547 genes to distinguish 22 human hematopoietic cell phenotypes; it defines activated MC as MCs activated by IgE receptors (24). Su et al. found fewer resting MCs in psoriatic lesions than in normal control samples (25). Zhang et al. found that tyrosine kinase receptor (c-Kit) and tryptase double-positive activated MCs were enriched in psoriatic skin and were progressively reduced in number after etanercept treatment, while resting MCs were nearly absent in psoriatic lesions. The significant upregulation of the MC activator IL-1B and the downregulation of *FAM124B* and *FAM174B*, identified as the specific genes in resting MCs, further support the important role of MC activation in the progression of psoriasis. In addition, a decrease in the total number of MCs and an increase in the proportion of activated MCs in psoriatic lesions have been reported (26). However, Liu et al. used the CIBERSORT method to evaluate immune cell infiltration based on hypermethylated genes in psoriasis and found a significant increase in resting MCs (27). The difference in results between studies may be related to the different stages of psoriatic lesions in the datasets. Gong et al. did not detect a difference in activated MCs between severe psoriasis and mild psoriasis, while resting MCs were decreased and correlated with disease severity (28). The authors suggested that this may be related to the fact that the gene expression profile used by the CIBERSORT algorithm for deconvolution mainly reflects IgE-activated MCs, while MC activation in psoriasis may be mediated by a non-IgE-dependent pathway.

## Contribution of mast cells to the immunopathogenesis of psoriasis

### T cells

The role of T cells in psoriasis is well-established. The functional interactions between MCs and T cells have been confirmed by several studies. The local density and activation of MCs are increased in a variety of T cell-mediated inflammatory diseases, such as rheumatoid arthritis, multiple sclerosis, and Crohn’s disease, and the co-localization of activated MCs and T cells has been reported in inflammatory tissues (29–32).

MCs show surface expression of major histocompatibility complex (MHC) I and II molecules that directly contact and activate T lymphocytes by interacting with T cell receptor (TCR), thereby inducing the antigen-specific clonal expansion of T cells (33, 34). Activated MCs act synergistically with IL-1β released in response to toll-like receptor (TLR)/FcϵRI receptor to enhance Th17 cell responses (35). In a hypercholesterolemic environment, MCs are capable of presenting Ags and effectively modulate CD4^+^ T effector cells to a proatherogenic Th1 phenotype within atherosclerotic plaques (36). IFN-γ-triggered mouse MCs can present Ags to CD4^+^ T cells, resulting in functional immunological synapses (37). Similarly, IFN-γ induces the expression of HLA-DR, HLA-DM, CD80, and CD40 on primary human MCs, which activate T cells by taking up and processing Ags and acting as antigen-presenting cells *in vitro* (38). In addition, the increased surface delivery of HLA class II and peptides induced by the degranulation of MCs further promotes T cell proliferation and creates a feed-forward loop of T cell-MC cross-activation (39).

MCs are a major source of IL-22 in patients with psoriasis (40). Gaudenzio et al. have shown that they act as inflammatory amplifiers in inflammatory lesions of patients with psoriasis. IFN-γ mediates the establishment of synaptic contacts between MCs and CD4^+^ T cells and induces their proliferation, leading to the induction of Th22 and IL-22^+^ IFN-γ^+^ Th cell subpopulations by the release of IL-6 and TNFα. Approximately 30% of unbound CD4^+^ T cells in psoriatic skin are IL-22^+^, while up to 60% of CD4^+^ T cells in contact with MCs express IL-22 (41). Mashiko et al. found that approximately 89% of c-Kit^+^ FcϵRI^+^ MCs in psoriatic lesions produced IL-22, whereas only about 1% of CD3^+^ T cells produced IL-22. c-Kit^+^ cells also produced IL-17, and most IL-17-producing c-Kit^+^ FcϵRI^+^ MCs co-expressed IL-22 (42).

MCs can transport contents, including micro-RNAs and MHC, to distant antigen-presenting cells *via* secretory extracellular vesicles. Phospholipase A2 (PLA_2_) is highly expressed in psoriatic lesions and produces cutaneous neolipid antigens for recognition by CD1a-reactive T cells. Cheung et al. found that the IFN-α-induced release of exosomes from MCs transferred PLA2 to adjacent CD1a hyper-expressing Langerhans cells, leading to the production of neolipid antigens and subsequent recognition by lipid-specific CD1a-reactive T cells, which induced the production of IL-22 and IL-17A (43).

It is currently believed that primary human MCs do not produce IL-17A by themselves; instead, exogenous IL-17A is captured and stored in specialized intracellular vesicles *via* receptor-mediated endocytosis. (44). Thus, in disease states, mast cells may function as sentinel cells by rapidly releasing preformed bioactive IL-17A to promote host defense and/or tissue inflammation. Eliasse et al. found that MCs are the primary source of IL-17 in early acne which relies on contact between activated CD4^+^ T cells and MCs, while in the post-inflammatory phase, neutrophils are recruited and amplify the production of IL-17 (45). Chen et al. demonstrated that synovial tissue-resident MCs in peripheral spondyloarthritis can act as IL-17A-loaded sentinel cells that release IL-17A to exacerbate tissue inflammation (46).

Recent studies have shown that neutrophil extracellular traps (NETs) play an important role in psoriasis. Lambert et al. have identified the connection between NETs and Th17 responses in psoriasis. Mutations in the *TRAF3IP2* gene in psoriasis promotes the ability of NET to induce Th17 cells in peripheral blood mononuclear cells and the secretion of IL-17A (47). Abundant polymorphonuclear neutrophils can release RNA, DNA, and cathelicidin (LL-37) *via* NETs to form nucleic acid–LL37 complexes, contributing to the release of TLR-mediated cytokines and chemokines; this also form a vicious cycle, promoting the release of NETs and exacerbating inflammation (48). As a chemoattractant for MCs, LL37 activates innate immune cells and disrupts innate tolerance to self-nucleic acids, resulting MC degranulation and the release of inflammatory mediators (49). In addition to neutrophils, several functions of other innate immune cells, such as MCs, macrophages, and eosinophils, in the form of extracellular traps (ETs) have gradually been revealed. The main components of MC extracellular traps (MCETs) are DNA, histones, and granule proteins, such as tryptase and LL-37. A variety of stimulants can induce MCET formation, such as IL-23 and IL-1β (50). MCs can exert antimicrobial activity by the release of MCET (von 51). In human psoriatic lesions, IL-17 is released at low levels by MCETosis, and IL-1β and IL-23 can rapidly induce MC degranulation and MCETosis in human skin, leading to the release of IL-17 and other MC products (50).

In addition, MCs can be activated by micro-vesicles released by T cells, thereby generating a response at the site of inflammation without contact with T cells (52). MCs can take up activated T cell-derived micro-vesicles. Shefler et al. demonstrated that MC-derived IL-24 induces the phosphorylation of signal transducer and activator of transcription-3 (STAT3) in keratinocytes of psoriasis after treatment with T cell-derived micro-vesicles (53).

### Tregs

Skin-resident regulatory T cells (Tregs) plays an important role in the maintenance of immune homeostasis. Tregs are mostly composed of CD4^+^CD25^+^FoxP3^+^ T cells, which can control the activity of other effector immune cells and maintain autoimmune tolerance by direct contact or the secretion of suppressive cytokines, such as IL-10 and transforming growth factor (TGF)-β. The dysregulation of these cells is closely associated with chronic inflammatory diseases (54). Tregs control the progression of skin inflammation by inhibiting the infiltration of CD4^+^ T cells, which produce granulocyte-macrophage colony-stimulating factor (GM-CSF), in the skin (55). In patients with psoriasis, dermal Tregs are dysfunctional, characterized by an inability to inhibit the proliferation of normal CD4^+^CD25^-^ responder T cells (Tresps) (56). The treatment of psoriasis may increase the number and activity of Tregs (57).

The interaction between Tregs and MCs has a bidirectional nature and is critical for immunosuppression in various diseases. Activated MCs inhibit Treg function *via* the OX40/OX40L axis in an IL-6-dependent manner, thereby promoting Th17 cell differentiation (58). MC-derived chymase downregulates immune tolerance-related cytokines, such as IL-10, TGF-β1, and IL-17A, thereby inhibiting Treg function. Chymase inhibitors effectively reduce inflammation and result in significant increases in the expression of immune tolerance-related cytokines, FoxP3 and Tregs (59). IL-10 and TGF-β secreted by Tregs can downregulate FcϵRI expression in MCs, thereby inhibiting MC maturation and degranulation (60).

Bovenschen et al. observed that CD4 ^+^ CD25 ^+^ Foxp3 ^+^ Tregs were more frequently located in the dermis than in the epidermis in patients with plaque psoriasis (61). The Koebner phenomenon occurs at sites of skin barrier damage in patients with psoriasis, and the use of tape stripping to remove the stratum corneum provides an *in vivo* model for skin barrier damage (62). Suttle et al. used tape stripping to induce the Koebner isomorphic response on normal skin of patients with psoriasis and found a significant decrease in the number of FoxP3^+^ Tregs in biopsies and an decreased number of apparent morphological contacts between tryptase^+^ MCs and FoxP3^+^ Tregs within 3–7 d in the Koebner-positive group (63). Thus, MCs may play a role in the development of the isomorphic response by crosstalk with Tregs to regulate the expression of related inhibitory factors.

### Keratinocytes

A growing number of recent studies have demonstrated that keratinocytes in psoriasis interact with immune cells to form a feed-forward inflammatory loop. They act as a trigger and amplifier of the inflammatory response by recruiting and activating immune cells, thereby triggering local inflammation, which plays important roles in the initiation, maintenance, and relapse phases of psoriasis (64).

Stem cell factor (SCF), which binds to c-Kit, is critical for the development, differentiation, and survival of MCs as well as recruitment, maturation, and migration in tissues. Keratinocytes represent pivotal cellular sources that trigger MC maturation and function in the skin; therefore, the homeostasis of resident skin MCs may be regulated in part by epidermal keratinocytes. Elevated serum SCF levels and significant enrichment of SCF in lesional keratinocytes were found in patients with psoriasis (65). The skin microbiota can extend into the dermis and establish physical contact with various cells beneath the basement membrane. The large amount of lipoteichoic acid (LTA) contained in the cell wall of Gram-positive bacteria triggers the production of SCF from adjacent keratinocytes *via* the TLR signaling pathway, thereby recruiting and regulating MCs in the dermis (66). Cho et al. further found that the expression of SCF is increased in the epidermis of IMQ-induced psoriasis-like lesions. The stimulation of rhIL-17 significantly improved the secretion of SCF in HaCaT cells and positively regulated MC proliferation (67). On the other hand, dexamethasone-activated MCs can promote keratinocyte proliferation *via* keratinocyte growth factor (KGF) *in vitro*, thereby disrupting skin barrier homeostasis (68).

Stressed keratinocytes can initiate the activation and recruitment of immune cells by the release of alarmins, including thymic stromal lymphopoietin (TSLP) or IL-33 (69). It has been suggested that herpes simplex virus type 2 (HSV-2)-infected keratinocytes contribute to the MC-mediated antiviral host defense *via* the IL-33/ST2 axis (70). IL-33, a member of the IL-1 cytokine family, regulates MC apoptosis and proinflammatory mediator release by binding to the ST2 receptor. Chymotrypsin and tryptases secreted by MCs facilitate the production of shorter, more mature, and more active IL-33 (4, 71). After release, IL-33 can diffuse from the epidermis into the dermis, triggering ST2-positive cells, including MCs, since the gene expression levels of *IL-33* and histidine decarboxylase are significantly increased in the skin of patients with psoriasis (72, 73). The co-administration of IL-33 with the peptide substance P (SP) significantly increases the expression and secretion of TNF and VEGF in MCs, further promoting angiogenesis and increasing inflammation in psoriasis (73, 74). TSLP is a pro-allergic cytokine expressed by keratinocytes and elevated in psoriatic lesions. MCs regulate TSLP production in keratinocytes *via* the tryptase/PAR-2 axis and are thereby involved in the development of inflammatory skin diseases (75). Vasoactive intestinal peptide (VIP)-positive nerve fibers in the dermis, dermal-epidermal junction, and epidermis are higher in patients with psoriasis than in undamaged and healthy skin. Th1 cytokines (i.e., TNF-α and IFN-γ) and VIP released by MC in the dermis induce the production of IL-6, SCF, and VEGF by keratinocytes, resulting in increased vascular permeability and amplification of the inflammatory condition of the skin (76).

### Adipocytes

The neuro-endocrine-immune system is an integrated regulatory network with an important role in organismal homeostasis. A complex triangular relationship among adipocytes, sympathetic nerves, and immune cells has been described (77). Neuro-immuno-metabolism is a promising but mysterious new area in the pathogenesis of psoriasis.

The sympathetic nervous system releases norepinephrine locally in adipose tissue (AT), thereby promoting lipolysis, and immune cells within adipose tissue are involved in the pathophysiology of obesity and its comorbidities. Innate immune cells promote insulin resistance (IR) in AT, which can act as a large secretory organ, producing substantially amounts of pro-inflammatory cytokines and adipokines that recruit relevant immune cells to drive systemic inflammation. AT-resident cells are significantly altered during obesity, with the accumulation of neutrophils, pro-inflammatory M1 macrophages, and MCs but a reduction in some cell types, e.g., Th2, Treg, and eosinophils (78). Some researchers have reported the accumulation of MCs in the AT of obese individuals. The number of MCs in subcutaneous AT is increased in individuals with metabolic syndrome and is positively correlated with IR and glucose levels (79, 80).

The effects of obesity on adipocytes mainly include the upregulation of pro-inflammatory adipokines, such as leptin and resistin, and the downregulation of anti-inflammatory adipokines, such as adiponectin (81). Obesity is one of the most common complications of psoriasis and an independent risk factor for the development of psoriasis (82). The levels of leptin and resistin are increased in patients with psoriasis and are positively correlated with disease severity, while the levels of lipocalin are decreased (83–85).

In a mouse model, IMQ-induced psoriasis-like skin inflammation was attenuated by a leptin deficiency, whereas leptin stimulation not only directly induced the pro-inflammatory phenotype in keratinocytes but also indirectly affected keratinocytes by inducing the secretion of pro-inflammatory cytokines from dermal fibroblasts (86, 87). Leptin can induce the production of IL-6 in keratinocytes by reducing the expression of caveolin-1 (88). MCs in AT are adjacent to leptin-producing adipocytes and are involved in the development of low-grade inflammation in obese individuals (89). A leptin deficiency in MCs facilitates the acquisition of an anti-inflammatory phenotype and polarizes macrophages toward M2 without affecting T cell differentiation (90). In addition, leptin can act as a paracrine or autocrine regulator of MC activity during inflammation by triggering the release of mediators, including cysteinyl leukotrienes (cysLTs) and chemokine CCL2 (91, 92).

Resistin exerts proinflammatory effects *via* the NF-kB signaling pathway by stimulating the production of cytokines, such as IL-6, IL-12, and TNF-α, thereby promoting the proliferation of keratinocytes and the recruitment of T cells to the skin and participating in the inflammatory process in psoriasis (93). Lipocalin has anti-inflammatory effects on keratinocytes *in vitro* by the inhibition of TNF-α. In psoriasis mouse models, a lack of lipocalin aggravates skin inflammation and the excessive infiltration of γδT cells producing IL-17 in the dermis (94). In addition, lipocalin induces the switch of MCs to an anti-inflammatory phenotype, mediating the production of IL-10 *via* the PI3K and p38 pathways, thus participating in the regulation of inflammation (91).

### Sensory neurons

It has recently been shown that in addition to roles in sensory signaling, sensory neurons are involved in the regulation of immune responses and associated inflammation *via* the secretion of various neuropeptides, such as SP, nerve growth factor (NGF), CGRP, VIP, and somatostatin. MCs can be activated by the stimulation of neuropeptides and the close anatomical proximity and functional communication of MC with sensory nerves has been detected in the intestine, skin, and respiratory tract (95, 96).

MC–neuron communication may contribute to the pathogenesis of non-allergic skin diseases, such as psoriasis and rosacea, and may play a critical role in the development of neurogenic inflammation, pain, and pruritus. In the skin, MCs enhance TRPV1 sensitivity or lower its activation threshold by releasing nociceptive and pruritogenic mediators, such as histamine, prostaglandins, and leukotrienes, and initiate reciprocal communication with specific nociceptors on sensory nerve fibers (97). Nerve fibers release neuropeptides to activate MCs by a feedback mechanism. In psoriatic lesions, the level of SP, nerve density, total number and degranulation of MCs, and contacts between MCs and sensory nerves are elevated (98). Nociceptors can drive IL-23-mediated psoriasis-like skin inflammation (99). Psoriasis is regulated by the brain–skin axis. The HPA axis is involved in the development of psoriasis by the release of CRH, ACTH, and glucocorticoids, and these regulate the skin response to stress and the topical immune response (100). MCs express CRHR1 in proximity of psoriatic plaques, and elevated CRH in psoriasis induces the degranulation of MCs. By contrast, the secretion of IL-6 by MCs can also induce the CRH secretion by the activation of the HPA axis by a feedback mechanism (101).

Mas-related G-protein-coupled receptors (Mrgprs) belong to the G protein-coupled receptor (GPCR) family and are mostly expressed by small-diameter sensory neurons in the peripheral nervous system. MRGPRX2 is a human Mrgpr and Mrgprb2 is the mouse ortholog of MRGPRX2. Human MCs are the only cells other than dorsal root ganglia that express MRGPRX2. MRGPRX2 can be activated by basic secretagogues, such as SP, somatostatin, compound 48/80, and mastoparan, which induce the activation of MCs by an IgE-independent pathway (102, 103). In the skin, MCs activated by MrgprB2/X2 can communicate locally with nearby peripheral neurons by releasing granules (104). IL-33 activates the JNK, p38, and NF-κB pathways in cutaneous MCs and synergistically stimulates the release of IL-8, TNF- α, CCL1, and CCL2 from MCs with MRGPRX2 (105). Bidirectional communication between MCs and neurons initiates and potentially increases inflammation as well as pruritus and pain perception in psoriasis. A better understanding of these interactions and the development of high-affinity MRGPRX2 inhibitors hold the promise of unlocking a new generation of therapeutic targets for neurogenic inflammation and non-histamine pruritus complicated by psoriasis.

## Conclusion

**Figure 1:**
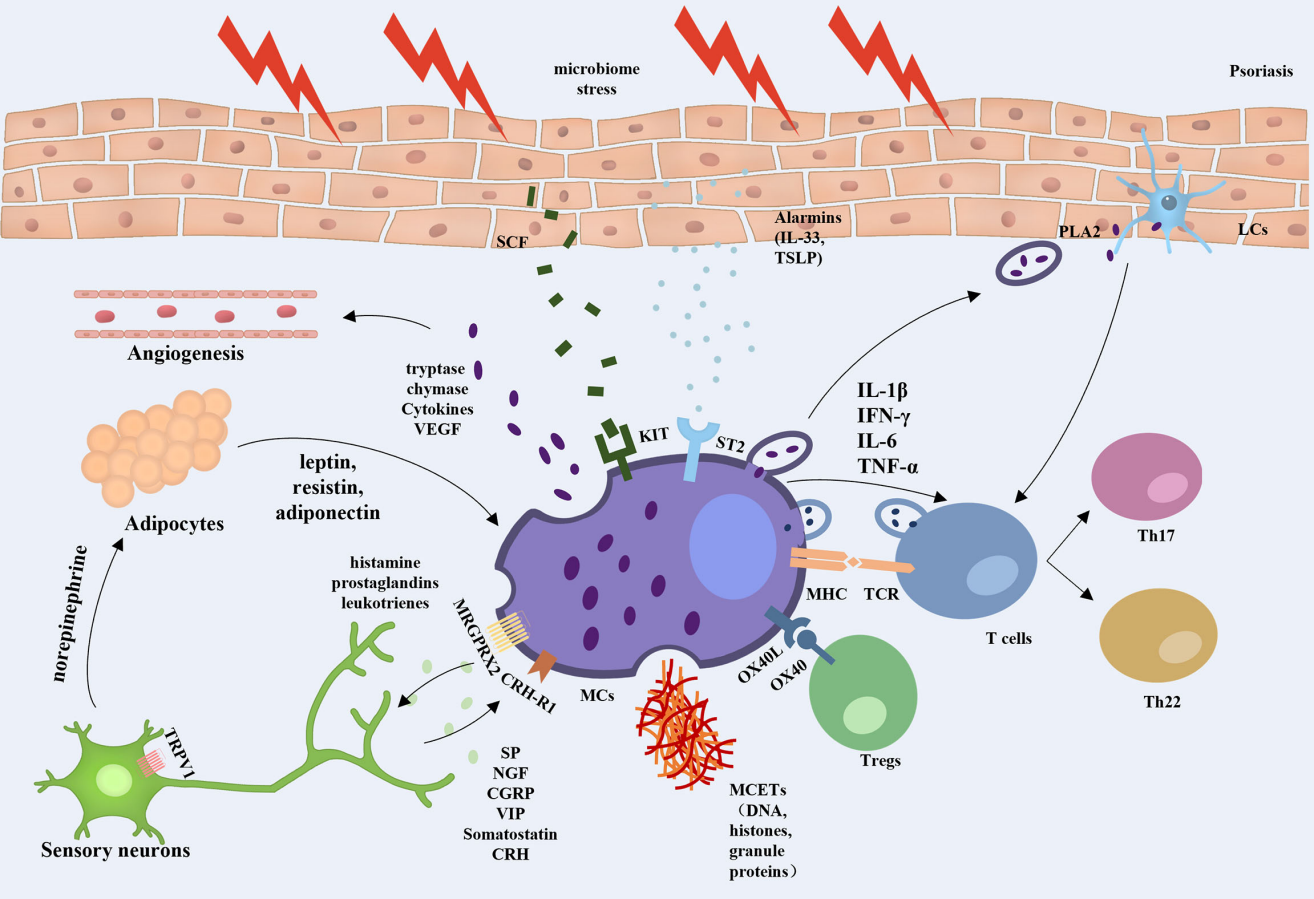
Overview of mast cell involvement in psoriasis. When exposed to external stresses, stimulated keratinocytes can promote angiogenesis and amplify inflammation by releasing alarmins to trigger ST2-positive MCs secreting TNF and VEGF, etc. The skin microbiota induces keratinocytes to produce more SCF, which bind to the c-kit and are involved in the recruitment and regulation of dermal MCs. MCs can activate T lymphocytes and strengthen Th17 and Th22 responses by interaction with TCR, secretion of extracellular vesicles, and formation of MCETs. MCs also regulate Treg function through the OX40/OX40L axis. Activated MCs were found to increase the sensitivity of TRPV1 in peripheral neurons by releasing nociceptive and pruritogenic mediators. While the stimulated nerve fibers release CRH and neuropeptides, which induce the activation of MCs *via* CRH-R1 and MRGPRX2 on the MC surface in a feedback manner. In addition, nerve fibers release norepinephrine into adipose tissue to regulate the secretion of leptin, resistin, and lipocalin in adipocytes, which promote the conversion of MCs to a proinflammatory phenotype, thus constituting a neuroendocrine-immune loop that is involved in the development of psoriasis.

In this review, we highlight the key role of MCs in psoriasis. MCs are involved in the initiation, development, and maintenance phases of psoriasis, with clear changes in the number and function of these cells, especially in the early stages of psoriasis. As a bridge between innate and adaptive immunity, MCs are involved in the regulation of inflammation and immune homeostasis in psoriasis by interacting with a variety of cells, including T cells, Tregs, neutrophils, DCs, keratinocytes, adipocytes, and sensory nerve cells, forming a complex cellular network (**Figure 1**).

Although our understanding of the role of MCs in the pathogenesis of psoriasis has improved considerably, relatively little is known about the mechanisms by which various factors regulate the specific functions of MCs. Studying the mechanisms underlying MC-immune cell interactions may provide a basis for restoring MC homeostasis and contribute to the treatment of psoriasis.

## Author contributions

X-YZ, J-AZ and KC contributed to conception and design of the study. X-YZ wrote the first draft of the manuscript. J-AZ and KC wrote sections of the manuscript. All authors contributed to manuscript revision, read, and approved the submitted version.

## Funding

This study is supported by grants from National Natural Science Foundation of China (No. 82273552, 82203947 and 82073445), Natural Science Foundation of Jiangsu Province (No. BK20210049), CAMS Innovation Fund for Medical Sciences (CIFMS-2021-I2M-1-001) and Medical Scientific Research Project of Jiangsu Provincial Health Commission (M2022113).

## Conflict of interest

The authors declare that the research was conducted in the absence of any commercial or financial relationships that could be construed as a potential conflict of interest.

## Publisher’s note

All claims expressed in this article are solely those of the authors and do not necessarily represent those of their affiliated organizations, or those of the publisher, the editors and the reviewers. Any product that may be evaluated in this article, or claim that may be made by its manufacturer, is not guaranteed or endorsed by the publisher.
